# Supplementary figures and images for: Two-Target Quantitative PCR To Predict Library Composition for Shallow Shotgun Sequencing
Source: mSystems. 2021 Jul 13;6(4):e00552-21. doi: 10.1128/mSystems.00552-21 (PMC8409737; doi:10.1128/mSystems.00552-21)

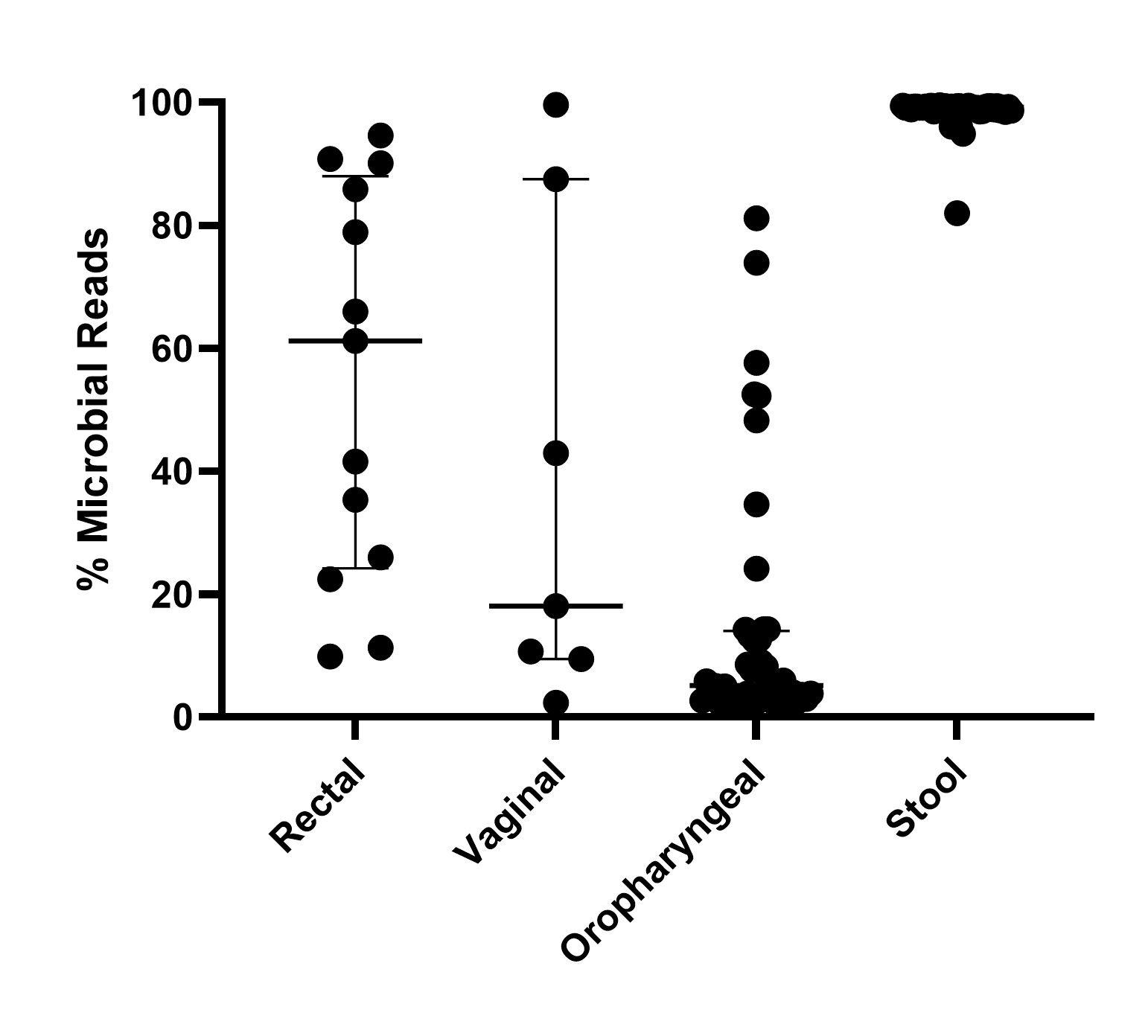

Supplement: FIG S1 [file msystems.00552-21-sf001.tif]

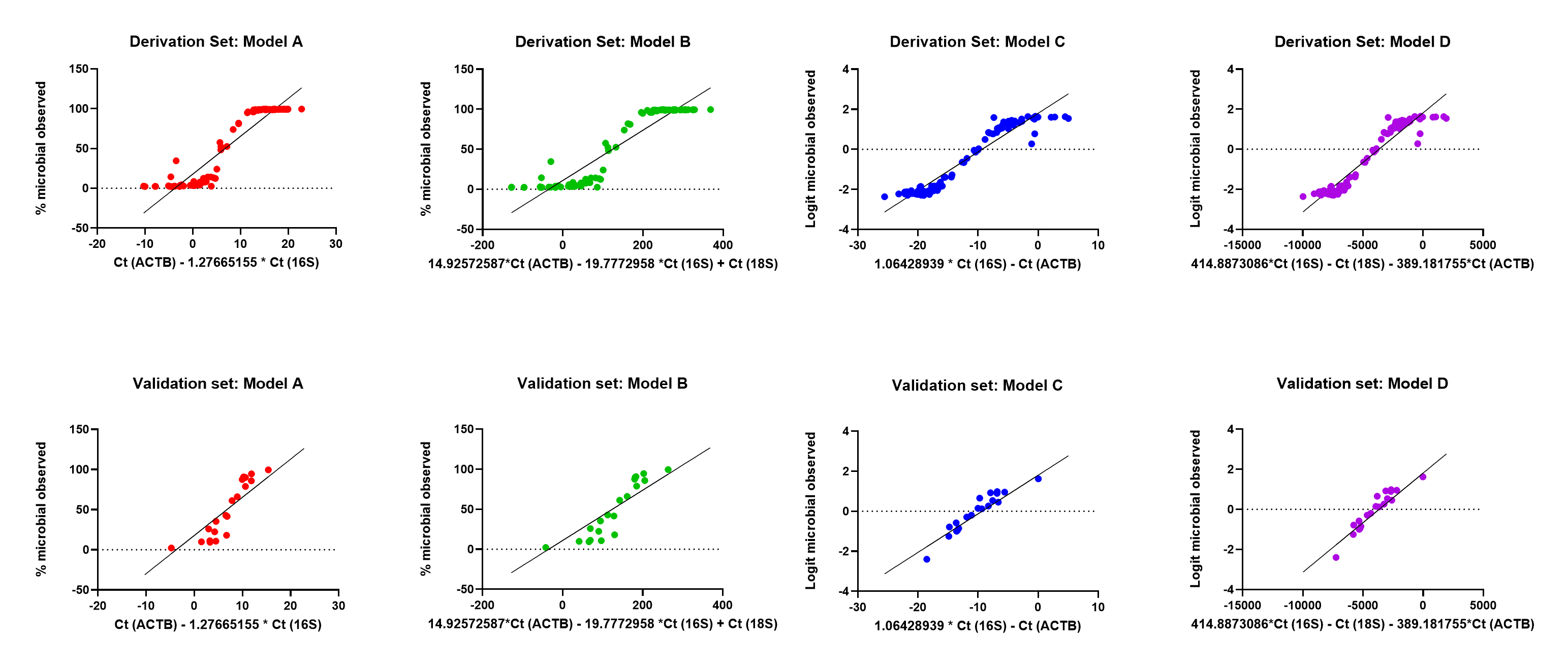

Supplement: FIG S2 [file msystems.00552-21-sf002.tif]

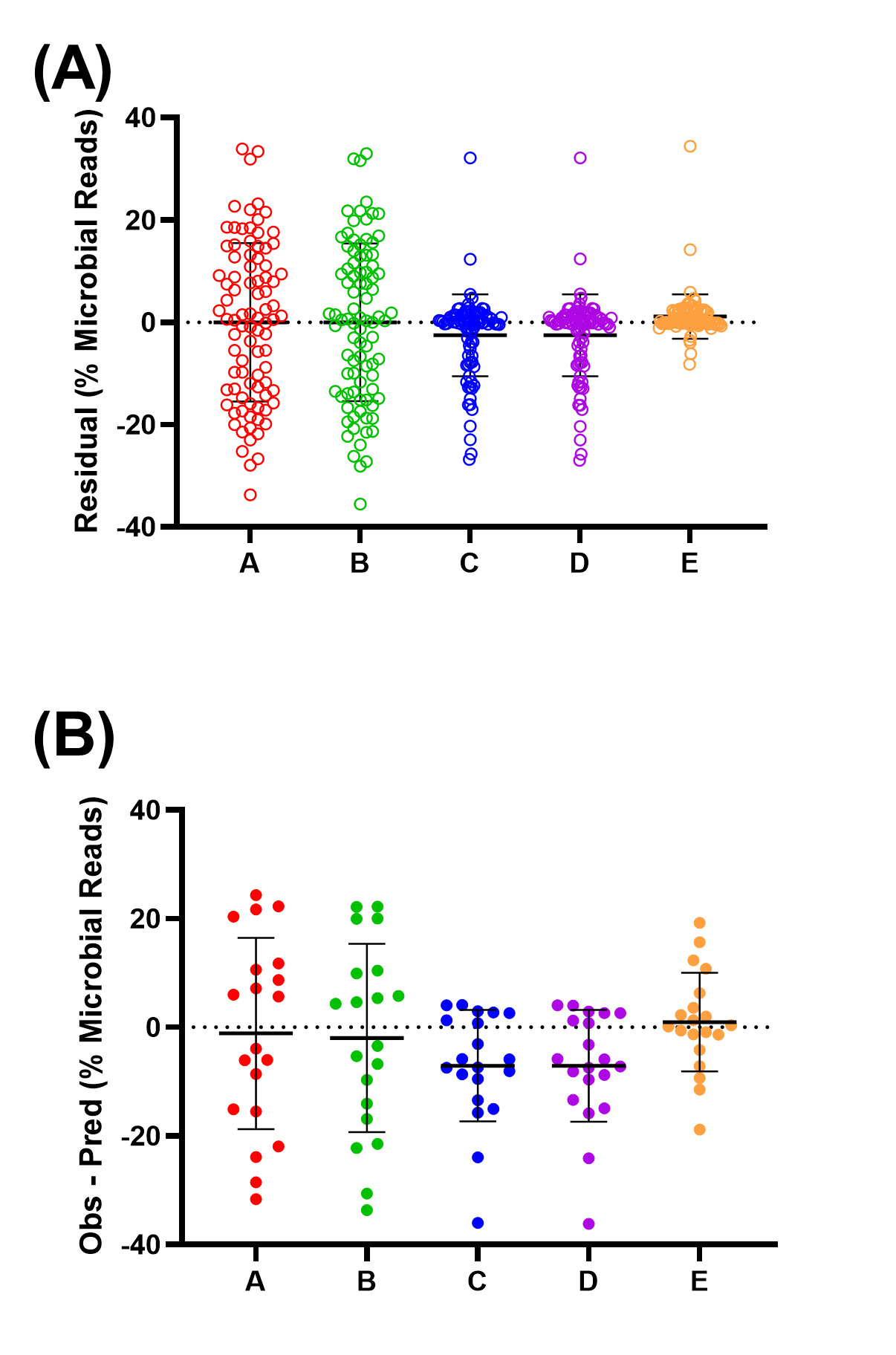

Supplement: FIG S3 [file msystems.00552-21-sf003.tif]

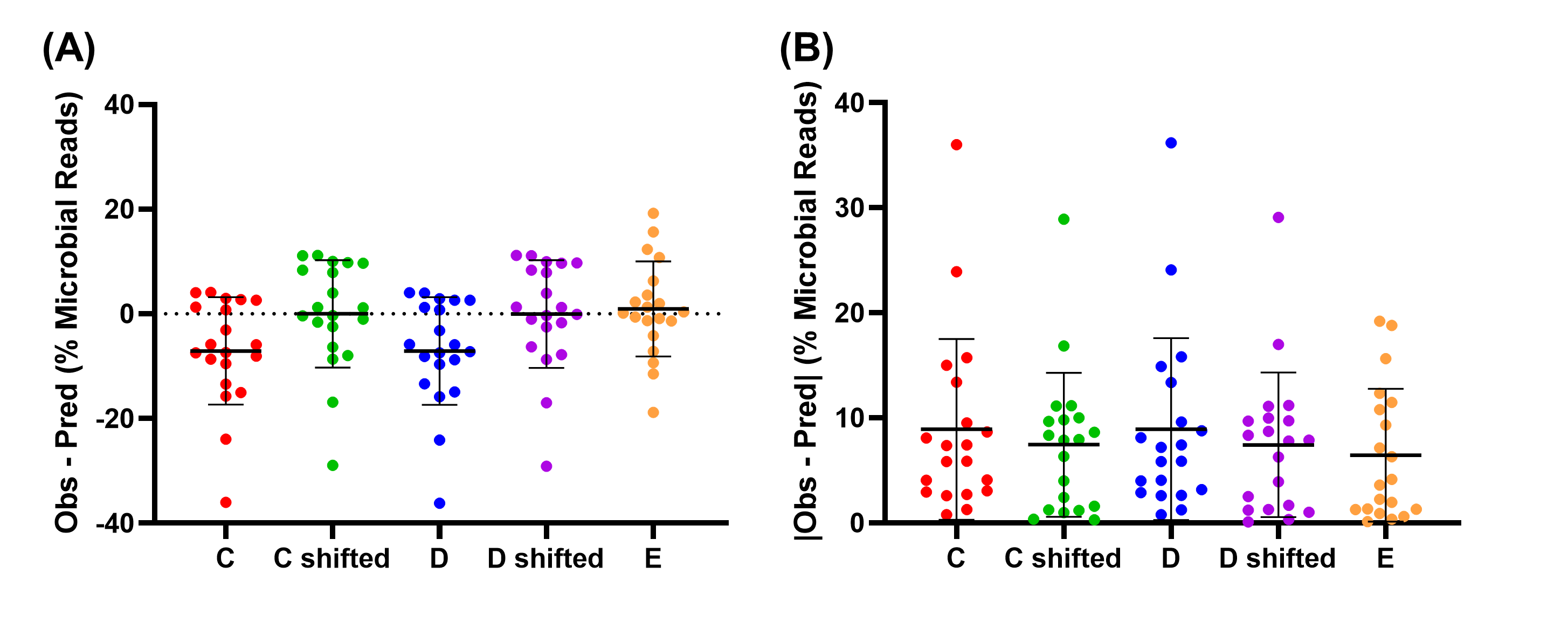

Supplement: FIG S4 [file msystems.00552-21-sf004.tif]
