# Supplementary material for: Two-Target Quantitative PCR To Predict Library Composition for Shallow Shotgun Sequencing
Source: mSystems. 2021 Jul 13;6(4):e00552-21. doi: 10.1128/mSystems.00552-21 (PMC8409737; doi:10.1128/mSystems.00552-21)
Supplement: TABLE S1 [file msystems.00552-21-st001.pdf]

(A)

Model	Range	Median	Mean	SD	Model type	qPCR data used
A	67.56	0.622	-3.371E-5	15.46	Linear regression	Human, bacterial
B	68.50	0.498	-4.494E-5	15.42	Linear regression	Human, bacterial, fungal
C	58.93	-0.099	-2.525	8.018	Linear regression after logit transformation of data	Human, bacterial
D	59.07	-0.097	-2.530	8.031	Linear regression after logit transformation of data	Human, bacterial, fungal
E	42.61	0.137	1.14	4.351	Sigmoidal model based on logistic growth curve	Human, bacterial

(B)

Model	Range	Median	Mean	SD
A	55.96	0.851	-1.130	17.61
B	55.88	-0.458	-1.962	17.33
C	40.10	-6.633	-7.074	10.26
D	40.27	-6.536	-7.110	10.28
E	38.03	0.248	0.944	9.101
