# Supplementary material for: Two-Target Quantitative PCR To Predict Library Composition for Shallow Shotgun Sequencing
Source: mSystems. 2021 Jul 13;6(4):e00552-21. doi: 10.1128/mSystems.00552-21 (PMC8409737; doi:10.1128/mSystems.00552-21)
Supplement: TABLE S2 [file msystems.00552-21-st002.pdf]

Target Gene	Forward Primer	Reverse Primer	Probe	Reference
**16S**	5-TCCTACGGGAGGCAGCAGT-3	5-GGACTACCAGGGTATCTAATCCTGTT-3	(FAM)-CGTATTACCGCGGCTGCTGGCAC-(NFQ-MGB)	Nadkarni *et al.* (2002) (6)
**18S (fungal)**	5-GGRAAACTCACCAGGTCCAG-3	5-GSWCTATCCCCAKCACGA-3	(56FAM)-TGGTGCATGGCCGTT-(NFQ-MGB)	Liu *et al.* (2012) (7)
**Human β-actin (ACTB)**	5-CGGCCTTGGAGTGTGTATTAAGTA-3	5-TGCAAAGAACACGGCTAAGTGT-3	(5VIC)-TCTGAACAGACTCCCCATCCCAAGACC-(3QSY)	Hasan *et al.* (2016) (8)
