# Supplementary material for: Two-Target Quantitative PCR To Predict Library Composition for Shallow Shotgun Sequencing
Source: mSystems. 2021 Jul 13;6(4):e00552-21. doi: 10.1128/mSystems.00552-21 (PMC8409737; doi:10.1128/mSystems.00552-21)
Supplement: TABLE S3 [file msystems.00552-21-st003.pdf]

Sample ID	Sample type	16S qPCR	18S qPCR	ACTB qPCR	Sequence Data Available
AH-PS-001	Rectal swab	17.150	37.014	24.881	Yes
AH-PS-003	Rectal swab	13.208	31.931	27.498	Yes
AH-PS-004	Rectal swab	13.772	38.475	29.418	Yes
AH-PS-006	Rectal swab	15.664	38.372	24.227	Yes
AH-PS-011	Rectal swab	13.859	37.765	26.632	Yes
AH-PS-016	Rectal swab	14.071	35.394	29.838	Yes
AH-PS-018	Rectal swab	17.716	39.907	27.141	Yes
AH-PS-020	Rectal swab	19.196	32.724	26.005	Yes
AH-PS-021	Rectal swab	15.276	36.371	27.362	Yes
AH-PS-031	Rectal swab	15.070	37.499	26.048	Yes
AH-PS-039	Rectal swab	16.950	33.237	24.876	Yes
AH-PS-044	Rectal swab	14.181	36.477	28.721	Yes
AH-PS-051	Rectal swab	15.744	41.958	30.329	Yes
HSV-11	Vaginal sample	15.288	42.211	29.487	Yes
HSV-16	Vaginal sample	14.883	45	34.364	Yes
HSV-24	Vaginal sample	13.352	24.588	23.588	Yes
HSV-29	Vaginal sample	16.097	40.941	25.045	Yes
HSV-31	Vaginal sample	14.188	39.880	24.816	Yes
HSV-32	Vaginal sample	16.705	29.166	24.585	Yes
HSV-8	Vaginal sample	22.317	45	23.727	Yes
S01	Stool sample	17.795	35.855	37.971	Yes
S03	Stool sample	12.796	33.182	32.530	No
S04	Stool sample	15.953	41.150	34.286	Yes
S05	Stool sample	16.624	32.771	35.094	No
S06	Stool sample	11.706	35.330	31.940	No
S07	Stool sample	16.193	41.100	33.827	Yes
S08	Stool sample	15.171	37.234	37.238	Yes
S09	Stool sample	12.662	30.542	35.180	No
S10	Stool sample	13.959	32.657	33.073	No
S11	Stool sample	14.976	34.039	36.013	Yes
S12	Stool sample	12.513	30.822	35.143	No
S13	Stool sample	17.762	36.456	38.448	Yes
S14	Stool sample	13.673	40.479	34.234	Yes
S15	Stool sample	15.140	31.801	32.067	Yes
S16	Stool sample	14.009	40.939	36.178	No
S17	Stool sample	14.019	34.625	34.945	Yes
S18	Stool sample	13.479	36.942	33.873	Yes
S19	Stool sample	18.192	37.932	38.773	Yes
S21	Stool sample	15.249	35.448	34.338	Yes
S22	Stool sample	11.641	32.887	32.052	Yes
S23	Stool sample	15.561	38.540	39.755	Yes
S24	Stool sample	12.515	31.872	34.281	Yes
S25	Stool sample	14.829	32.765	36.232	Yes
S26	Stool sample	12.113	32.025	32.639	No
S27	Stool sample	11.062	36.782	33.921	No
S28	Stool sample	11.023	31.031	32.774	Yes

S29	Stool sample	13.702	34.043	31.340	Yes
S30	Stool sample	12.871	29.518	30.345	Yes
S31	Stool sample	13.616	38.314	28.816	Yes
S32	Stool sample	13.472	32.782	28.768	No
S33	Stool sample	14.442	37.292	33.206	No
S34	Stool sample	11.389	28.368	29.994	Yes
S35	Stool sample	11.432	34.383	33.811	Yes
S36	Stool sample	10.478	38.662	33.306	Yes
S37	Stool sample	16.335	36.186	36.116	Yes
S38	Stool sample	11.495	34.770	29.953	Yes
S40	Stool sample	12.412	39.737	29.455	Yes
S41	Stool sample	12.646	40.869	28.918	Yes
S42	Stool sample	13.944	37.592	32.277	Yes
S43	Stool sample	14.506	40.968	31.082	Yes
S44	Stool sample	13.010	37.683	39.400	Yes
S45	Stool sample	14.115	31.804	35.008	No
S46	Stool sample	11.742	30.659	29.168	Yes
S47	Stool sample	12.069	22.504	35.036	Yes
S48	Stool sample	12.925	28.786	26.093	Yes
S49	Oropharyngeal sample	22.074	38.849	24.316	Yes
S50	Oropharyngeal sample	22.219	37.845	34.049	Yes
S51	Oropharyngeal sample	21.962	39.376	30.278	Yes
S52	Oropharyngeal sample	19.334	38.227	30.600	Yes
S53	Oropharyngeal sample	24.129	38.458	22.908	Yes
S54	Oropharyngeal sample	26.864	39.046	29.193	Yes
S55	Oropharyngeal sample	19.412	35.294	27.238	Yes
S56	Oropharyngeal sample	18.957	40.031	30.068	Yes
S57	Oropharyngeal sample	20.471	36.821	26.683	Yes
S58	Oropharyngeal sample	25.027	38.536	29.333	Yes
S59	Oropharyngeal sample	22.298	38.109	28.679	Yes
S60	Oropharyngeal sample	26.424	38.576	25.954	Yes
S61	Oropharyngeal sample	17.499	37.829	26.106	No
S62	Oropharyngeal sample	21.777	36.727	27.643	Yes
S63	Oropharyngeal sample	20.885	39.852	22.967	Yes
S64	Oropharyngeal sample	14.190	34.514	27.726	No
S65	Oropharyngeal sample	21.052	38.109	28.394	No
S66	Oropharyngeal sample	18.724	38.983	26.727	Yes
S67	Oropharyngeal sample	21.598	31.545	27.956	Yes
S68	Oropharyngeal sample	16.442	37.661	28.150	Yes
S69	Oropharyngeal sample	25.848	39.483	23.016	Yes
S70	Oropharyngeal sample	25.835	42.342	30.654	Yes
S71	Oropharyngeal sample	16.629	18.785	21.802	No
S72	Oropharyngeal sample	19.454	38.747	27.179	No
S73	Oropharyngeal sample	23.210	27.757	25.161	Yes
S74	Oropharyngeal sample	21.070	38.235	24.119	Yes
S75	Oropharyngeal sample	15.914	38.314	28.776	Yes
S76	Oropharyngeal sample	21.559	37.893	24.027	Yes

S77	Oropharyngeal sample	20.904	38.373	29.820	No
S78	Oropharyngeal sample	20.763	36.163	27.838	No
S79	Oropharyngeal sample	19.000	30.308	24.625	Yes
S80	Oropharyngeal sample	20.194	38.671	27.235	Yes
S81	Oropharyngeal sample	22.237	40.811	28.746	Yes
S82	Oropharyngeal sample	21.980	39.41	26.133	Yes
S83	Oropharyngeal sample	24.245	32.442	26.367	Yes
S84	Oropharyngeal sample	16.492	40.227	24.917	Yes
S85	Oropharyngeal sample	17.823	38.023	27.809	Yes
S86	Oropharyngeal sample	19.597	29.21	25.971	Yes
S87	Oropharyngeal sample	17.504	38.249	26.702	No
S88	Oropharyngeal sample	23.070	37.249	24.581	No
S89	Oropharyngeal sample	16.545	35.363	25.871	Yes
S90	Oropharyngeal sample	20.237	35.45	27.512	Yes
S91	Oropharyngeal sample	20.810	36.607	26.057	Yes
S92	Oropharyngeal sample	24.735	43.749	21.284	Yes
